# Functionalized Gold Nanorods for Tumor Imaging and Targeted Therapy

**DOI:** 10.7497/j.issn.2095-3941.2012.04.002

**Published:** 2012-12

**Authors:** Chen Gui, Da-xiang Cui

**Affiliations:** Department of Bio-Nano Science and Engineering, Key Laboratory for Thin Film and Microfabrication of the Ministry of Education, Institute of Micro-Nano Science and Technology, Shanghai Jiaotong University, Shanghai 200240, China

**Keywords:** gold nanorods, surface functionalization, tumor, molecular imaging, photothermal therapy

## Abstract

Gold nanorods, as an emerging noble metal nanomaterial with unique properties, have become the new exciting focus of theoretical and experimental studies in the past few years. The structure and function of gold nanorods, especially their biocompatibility, optical property, and photothermal effects, have been attracting more and more attention. Gold nanorods exhibit great potential in applications such as tumor molecular imaging and photothermal therapy. In this article, we review some of the main advances made over the past few years in the application of gold nanorods in surface functionalization, molecular imaging, and photothermal therapy. We also explore other prospective applications and discuss the corresponding concepts, issues, approaches, and challenges, with the aim of stimulating broader interest in gold nanorod-based nanotechnology and improving its practical application.

## Introduction

Gold nanorods (GNRs) with distinctive shape-dependent optical properties have drawn worldwide attention in the biomedical field. These nanorods possess two different plasmon bands. One is the transverse plasmon band in the visible region at around 520 nm, and the other is the longitudinal plasmon band in the near-infrared region. These unique properties make gold nanorods excellent materials for biological sensing, imaging, photothermal therapy, drug delivery, and other biological applications^[^^1^^-^^4^^]^. However, there are several problems that influence and limit the application of gold nanorods. A small change in the size, shape, local environment, surface nature, and degree of aggregation of nanorods will lead to tunable changes in their properties, which in turn affect their applications^[^^5^^]^.

GNRs have three main advantages^[^^6^^]^: (1) the SPR extinction in the near-infrared (NIR) region of GNRs provides opportunities in NIR photoabsorption and scattering and medical photothermal therapy; (2) the stronger longitudinal SPR band makes GNRs more sensitive to changes in size, shape, nano-environment, or interparticle distance; (3) the tunable SPR band can improve surface-enhanced Raman scattering sensing and imaging. Based on these merits, GNRs can be widely used in many medical applications, including biosensing, drug delivery, photothermal therapy, and imaging.

GNRs are usually produced by the simple seed-mediated approach and surfactant cetyltrimethyl ammonium bromide (CTAB) directed synthesis^[^^7^^,^^8^^]^, which both produce a high yield of homogeneous gold nanorods with LSPR resonances in the visible and near-infrared. A large amount of CTAB is used in the CTAB synthesis. CTAB is not only the structure-directing agent of anisotropic growth, but also the stabilizer that forms bilayers on the gold nanorods^[^^9^^]^. After the reaction, some CTAB remains on the surfaces of the nanorods^[^^10^^]^. However, the large amounts of CTAB existing in the solution interfere with the biological processes and exhibit high cytotoxicity^[^^11^^-^^13^^]^. Alaaldin et al.^[^^13^^]^ found that all CTAB-capped gold nanorod solutions display significant cytotoxicity at a certain concentration (65% to 75% loss of viability). Moreover, the CTAB on the surface of gold nanorods cannot be easily replaced with biomolecules. Removing CTAB from the surface may cause the uncontrollable aggregation of gold nanorods. The toxicity is not caused by the CTAB layers that are statically bound to the nanoparticles, as Connor et al.^[^^14^^]^ quantitatively discussed in their paper. These researchers reported that 1 µm of CTAB-passivated nanoparticles did not exhibit cytotoxicity to cultivated cells after three times of centrifugation, whereas the unbound CTAB was found to be toxic to cultivated cells. Thus, excess CTAB should be removed from nanorods. Several studies have indicated that repeated centrifugation decreases excess CTAB^[^^15^^]^. However, the CTAB bilayers still exist on the surface of gold nanorods after the centrifugation. These bilayers can be desorbed from the surface. However, the elimination of the desorbed CTAB would lead to the aggregation of gold nanorods.

A number of studies have focused on surface modification of gold nanorods to address the abovementioned issues. Surface functionalization can provide stability, solubility, and retention of optical properties in various media; it can also reduce cytotoxicity. Thus, it remains a key step in the synthesis of GNRs. For example, Zhang et al.^[^^16^^]^ found that the use of polyamidoamine (PAMAM) to modify gold nanorods greatly increased biocompatibility and reduced cytotoxicity. **Table 1** presents a comprehensive comparison of the cytotoxicity of nude CTAB-GNRs and surface-modified GNRs.

**Table 1:** Cytotoxicity of CTAB-GNRs and modified GNRs.

Cytotoxicity	CTAB-gold nanorods	Modified gold nanorods
Cell viability to HT-29 cells	0.4 nM CTAB-GNRs:30% or so	0.4 nM PAA-GNRs: 90% or so
		0.4 nM PAH-GNRs: >80%
Cell viability to HeLa cells	0.09 mM CTAB-GNRs:>80%	0.09 mM PC-GNRs: >90%
	0.18 mM CTAB-GNRs: 70% or so	0.18 mM PC-GNRs: >90%
	0.36 mM CTAB-GNRs: 40% or so	0.36 mM PC-GNRs:>90%
	0.72 mM CTAB-GNRs: 20% or so	0.72 mM PC-GNRs:>90%
	1.45 mM CTAB-GNRs: hardly any cells survived	1.45 mM PC-GNRs: 80% or so
Cellular uptake by HT-29 cells	0.2 nM CTAB-GNRs: 45±6 nanorods per cell	0.2nM PAA-GNRs: 270±20 nanorods per cell
		0.2 nM PAH-GNRs: 2,320±140 nanorods per cell
Cellular uptake by MCF-7	CTAB-GNRs (GNR=1.1):9,069±2,171 nanorods per cell	PSS-GNRs: 3,484±700 nanorods per cell
		PDDAC-GNRs: 117,519±12,496 nanorods per cell

The above data were obtained from these references [13, 17, 18].

There are two common functionalization strategies. One strategy is the ligand exchange of original surfactants with hydrophilic ligands. The other strategy is to form an interdigitated bilayer between the amphiphilic molecules or polymers and the passivating surfactant layer on the surface of nanoparticles^[^^19^^]^. However, some challenges still exist. In Bushnu’s report^[^^20^^]^, the direct exchange of CTAB for a functional thiol could not be facilitated. The GNRs synthesized through the seed-mediated method are hydrophilic. Many thiols are not soluble in water and their dispersion in an aqueous solution of CTAB-stabilized nanorods would not result in ligand exchange. However, the direct dropwise addition of a concentrated solution of 4-mercaptophenol in THF into an aqueous growth solution of rods led to CTAB-thiol exchange and slow precipitation of phenol-functionalized nanorods. Dentili et al.^[^^21^^]^ utilized the double-phase transfer to functionalize GNRs.

Nowadays, alternative coating methods for gold nanorods include silica coating, layer-by-layer polyelectrolyte coating, chain transfer polymer absorption on the nanorod surface, amphiphilic block copolymer coating, phospholipid coating, polymer coating by interdigitated bilayer formation, and incorporation of nanorods into poly (N-isopropylacrylamide) gel or alginate gel. However, not all these methods are designed to prepare functional gold nanorods. A good coating should provide chemical functionality for conjugation chemistry, good water solubility, and colloidal stability during modification and purification steps^[^^22^^]^. Surface functionalization of nanoparticles is an important step to realize nano-bio-photonic applications.

In this paper, we review some of the main advances made over the past few years in the study of gold nanorods for surface functionalization, molecular imaging, and photothermal therapy. We also explore other prospective applications and discuss the corresponding concepts, issues, approaches, and challenges with the aim of stimulating broader interest in gold nanorod-based nanotechnology and improving its practical application.

## Surface Functionalization of Gold Nanorods

In recent years, the study of surface functionalization of gold nanorods has made great advances. For example, gold nanorods can now be organized into gold nanorod arrays by adding oligonucleotides; dendrimers can be used to replace the CTAB molecules on the surface of GNRs, PEG-modified GNRs can enhance biocompatibility of GNRs; and magnetic nanoparticles now coat the surface of GNRs. These advances are reviewed in this paper.

### DNA-templated ordered array of gold nanorods

**Figure 1:**
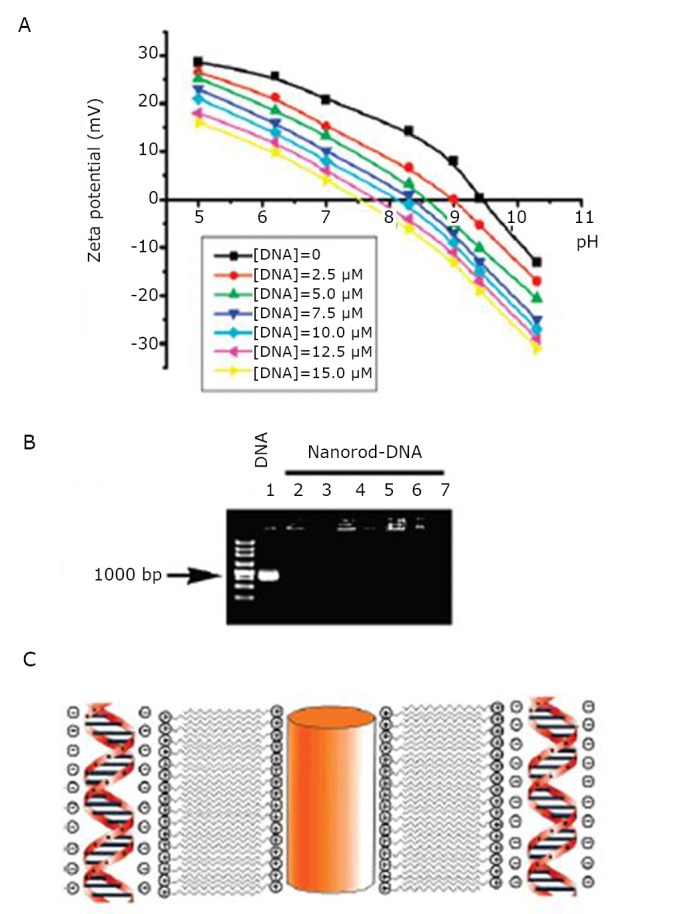
A: Potential of gold nanorods with an aspect ratio of 3.0 in a DNA solution with pH from 5.0 to 11.0. B: Agarose electrophoresis mobility retardation assays of DNA-nanorod complexes: (lane 1) 5M. DNA: (lanes 2-7) nanorod-DNA complexes with [DNA] of 2.5, 5.0, 7.5, 10.0, 12.5, and 15.0 íM, respectively. C: Cartoon illustrating “zipping”: the formation of the bilayer of CTAB on the nanorod surface and the complexation between nanorods and DNA. Reprinted with permission from [23], Pan B, Cui D, Ozkan C, et al. DNA-templated ordered array of gold nanorods in one and two dimensions. J Phys Chem C 2007; 111: 12572-12576.

Pan et al.^[^^23^^]^ utilized DNA as templates to allow the self-assembly of gold nanorods. Researchers found that GNRs could assemble into one- and two-dimensional structures under suitable conditions, such as appropriate aspect ratio and DNA concentration. The researchers also explained rod–rod self-assembly at different DNA concentrations by the ζ potential. In **Figure 1A**, the ζ potential is decreased by increasing DNA template concentration, indicating the existence of electrostatic interaction between the positively charged nanorod surface and the negatively charged DNA surface. The mobility of the complexes was completely retarded, indicating that the electrostatic attraction between the CTAB on the nanorods and the negatively charged DNA was extremely strong that DNA mobility was retarded in the sample wells (**Figure 1B**). Ternary DNA-CTAB-nanorod complexes were formed when the CTAB-coated gold nanorods were mixed directly with DNA (**Figure 1C**). The nanorod-DNA complexes were fully assembled by the electrostatic interaction between CTAB and DNA. These well-ordered, immobilized gold nanorods can be used in various applications such as optical and electronic devices, biosensors, and catalysis.

### Dendrimer modified gold nanorods

Dendrimers are a class of polymers with a highly ordered structure. Dendrimers coated on nanoparticles can alter the charge and reactivity as well as enhance the stability and dispersion of the nanoparticles. Li et al.^[^^24^^]^ selected the partially thiolated PAMAM dendrimer to replace the CTAB molecules on the surface of gold nanorods, thus improving the biocompatibility of the prepared GNRs. Cui et al.^[^^25^^]^ reported dendrimer-coated GNRs exhibited very low cytotoxicity, which could be useful in future tumor therapy.

### PEG-modified gold nanorods

Polyethylene glycol (PEG) modification is a common strategy to prepare biocompatible materials. Niidome et al.^[^^26^^]^ utilized PEG-modified gold nanorods. mPEG-SH was added to the CTAB solution, and the excess CTAB was removed by dialysis. The PEG-modification of gold nanorods was then confirmed by measuring the ζ potentials, which decreased to nearly the neural level. The results indicated that the PEG chain bound to the gold nanorods and the PEG-modified gold nanorods exhibited low cytotoxity. The PEG-modified gold nanorods were stable in the blood circulation with a half-life period of approximately 1 h. No accumulation was found in the major organs, except in the liver, for at least 72 h. PEG-modification was found to be an excellent alternative method to remove CTAB and stabilize nanorods in physiological conditions. PEG-modified gold nanorods were also found to have long lasting circulation in the blood.

Huang et al.^[^^4^^]^ also utilized PEGylated gold nanorods and demonstrated that gold nanorods could be maintained in extracellular space, which could be an effective way of inducing cell death in cancer cells.

Niidome T^[^^27^^]^ successfully prepared biocompatible PEG-coated gold nanorods and utilized them for photothermal cancer therapy. The growth of the tumor after the treatment was recorded. Only the case treated with both direct injection of gold nanorods and irradiation of laser light showed a significant suppression of the tumor. However, the control mice injected with gold nanorods or treated with laser irradiation showed no differences in tumor growth from the non-treated mice.

### Iron oxide-coated gold nanorods

A magnetic core-gold shell system can separate precursor materials via magnetism, unlike the inverse system. The magnetic core-gold shell system has a promising biomedical application. The hybrid nanomaterials combine optical and magnetic properties, making them potentially useful for biomedical application^[^^28^^]^.

Gole et al.^[^^29^^]^ proposed two methods to form iron-coated gold nanorods. One was a simple method where gold nanorods were produced through a three-step seed-mediated approach. The prepared gold nanorods were coated with a layer of poly (sodium 4-styrenesulfonate). The polymer with the negative charge attracted iron II and iron III in the solution. Then the iron salts began to form uniform coatings onto the surface of the GNRs. The other method utilized electrostatic interaction by combining iron oxide particles with the CTAB-coated gold nanorods to assemble the complex. The iron-coated gold nanorods formed through the first method exhibited better uniformity. However, the nanorods formed through the second method were more responsive to a magnetic field.

### Silica-coated gold nanorods

Amorphous silica nanoparticles can become compatible with biomolecules via physical absorption or electrostatic interaction^[^^11^^]^. Coating the surface of nanorods with silica can also preclude the aggregation of nanoparticles. Moreover, adjusting the thickness of the silica shell will affect the optical properties of the nanocomposites. Zhang et al.^[^^11^^]^ used a seed-mediated growth approach to synthesize GNRs. The gold nanorod core was surrounded by a silica layer via TEOS polymerization. The prepared core or shell nanocomposites had good solubility and biocompatibility. These properties were found to be useful for Hb immobilization and biosensor fabrication. The results showed that Hb retained its biological activity and displayed high stability, and the biosensor allowed the fast and direct electron transfer of Hb GICA.

Cong et al.^[^^30^^]^ confirmed the difficulty of forming a uniform silica shell on the surface of GNRs because of the high concentration of CTAB. To solve this problem, several researchers applied an intermediate material to the CTAB-coated gold nanorods. For instance, Chen et al.^[^^31^^]^ substituted CTAB with mPEG-thiol and then formed a silica shell with the silica-coupling agent mPEG polymer. Cong et al.^[^^30^^]^ found that as long as CTAB could be removed, a silica deposition with uniformity and high quality could also be obtained. In this study, a sufficient amount of CTAB remained, allowing the nanoparticles to provide adequate surface for silica functionalization. Controlling the amount of TEOS produced GNRs with silica shells of different thickness. For further analysis, the silica-coated nanorods were coated by a gold layer. After the procedure, the nanorods yielded a novel gold-silica-gold structure. Multilayer nanorods have unique optical properties that rely on the thickness of both the silica shell and the gold overcoat. Specifically, surface resonance can be widely tuned by changing the dimensions of the silica and the gold layers. The gold overcoat produces a broad plasmon band that is red-shifted by as much as 1000 nm, thus making the nanorod a convenient tool to enhance weak near-IR radiative transitions.

Silica-coated GNRs can be connected with DNA for enhanced surface plasmon resonance imaging (SPRI). Sendroiu et al.^[^^32^^]^ presented functionalized single-strand DNA silica-coated GNRs. These GNRs exhibit an intense absorption band at 780 nm and are used to enhance the sensitivity of SPRI measurements on DNA microarrays.

### Lipid-gold nanorod composites

Lipid bilayers are biocompatible and display reactive and interactive functionality. The facile formation of lipid bilayers onto solid surface has attracted much attention. Recently, several studies have focused on the combination of lipids and nanomaterials for bio-analytical applications. A limited number of methods for preparing lipid-capped GNRs have been reported to date. The supported bilayer easily interacts with hydrophilic surfaces, such as glass, mica, and silicon, thereby generating a conformal and almost flawless coating^[^^33^^]^. The lack of membrane-surface adsorption causes phospholipid vesicles to resist rupture and form the supported bilayers^[^^34^^]^. Recently, Cho et al.^[^^35^^]^ found that supported lipid bilayers can be formed in gold through the use of an amphipathic α-helical viral peptide.

Takahashi et al.^[^^36^^]^ proved that phosphatidylcholine controls the aggregation of gold nanorods. In their experiments, CTAB was removed from the gold nanorod solution and placed into a chloroform phase containing phosphatidylcholine (PC). The PC-gold nanorods exhibited low cytotoxity because PC is not toxic to living cells.

**Figure 2:**
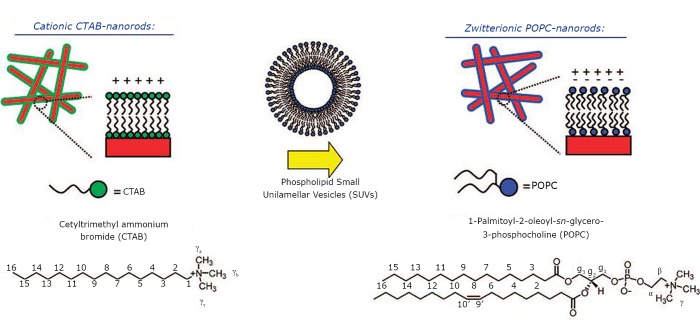
Modification process of GNRs. Reprinted with permission from [33], Orendorff CJ, Alam TM, Sasaki DY, et al. Phospholipid− Gold Nanorod Composites. ACS Nano 2009; 3: 971-983.

The team of Christopher J. Orendorff et al.^[^^33^^]^ utilized the vesicle fusion approach to form a lipid bilayer on gold nanorods and demonstrated its formation. The CTAB layer was replaced by POPC liposomes, and the excess was removed with the final concentration of 40 µL in 1 mL. **Figure 2** shows the modification process. The lipid-gold nanorod composites have zwitterionic functionality. Compared with as-prepared CTAB-gold nanorods, the POPC-modified composites were ~30 mv charge. The CTAB-gold nanorods self-assembled into disordered arrangements because of end-to-end or end-to-side electrostatic repulsion, whereas the lipid-gold nanorod composites assembled side-to-side in short-range order at pH 7.0. By decreasing pH, the lipid-gold nanorod structures would disassemble because of their cationic and repulsive surface potential.

Basiruddin et al.^[^^22^^]^ developed a reverse micelle-based polyacrylate coating for gold nanorods. The resultant core-shell structure nanorods could be applied to obtain various functional nanorods. The micelle-based coating was a robust coating because it produced water-soluble gold nanorods without shape change or particle aggregation. To successfully develop the coating, the aforementioned researchers summarized three key steps. First, in the reverse micelle solution, the nanorods and the polymer formed monomer precursors that were soluble. Polymerization took place in situ to minimize aggregation and shape change of nanorods. Second, the concentration of polymer should be appropriate to generate stable coating and achieve the desired functionality on the coating backbone. Finally, polymer-forming monomers were used in high concentration to replace the surfactant during polymeration. The reaction was halted before completion so that cross-linking between particles could be ignored. The coating method developed in this study could absorb a variety of chemical functionalities, such as primary amines, poly(ethylene glycol), and fluorescein, making these functionalities more suitable for biomedical application.

Castellana et al.^[^^37^^]^ used a thiol-gold linkage to tether some of the lipids to the GNRs and produced stable lipid-capped GNRs. The TEM images indicated that the lipid-capping layer protected the GNRs during the production of the biosensor and after multiple cycles of centrifugation and resuspension. They utilized the hybrid lipid-coated GNR biosensor to capture and detect membrane-active drugs.

This study underscores the potential of lipid-coated GNRs for biosensor applications. And the researchers offered some excellent ideas to further develop powerful multiplexed label-free sensor platforms. For example, they mentioned that tailoring the chemical and physical properties of the lipids by altering their composition or by attaching targeting molecules to the headgroups of the lipids would allow the incorporation of additional degrees of selectivity into the biosensor platform. This indicated that lipid-capped GNRs play an important role in biosensors.

### Other polymer-coated gold nanorods

Chitosan is a natural, biodegradable carbohydrate-based polymer widely used in water treatment, heavy metal removal, cosmetic additives, photographic paper, and other applications. Nandanan et al.^[^^19^^]^ used two new polymers to modify the surface of gold nanorods. Both polymers were derived from chitosan oligosaccharides, which contain multiple thiols or oiled acid, as well as primary amine groups. Gold nanorods easily aggregated after a complete ligand exchange with polymer I. Polymer II was employed as the coating for the hydrophilic gold nanorods, a procedure in partial ligand exchange. Fictionalization with polymer II was conducted by sonicating the nanorod-polymer mixture in water. This study also indicated that partial ligand exchange was necessary for the nanorods to achieve water solubility. The multiple thiol and oleyl groups in the backbone polymer provided numerous binding sites to the nanoparticles. The researchers also discovered that the polymer-coated gold nanorods could be further modified with biomolecules for applications such as biolabeling and biosensing.

Goodwin et al.^[^^38^^]^ utilized singly substituted conjugates of Dextran and a phospholipid (Dextran-DSPE) as stable coatings for nanoparticles. The suspensions with Dextran-DSPE yielded more nanorods than similar suspensions with PEG-phospholipid and commonly used thiol-PEG. Dextran-DSPE was found to be a potential surfactant material for the functionalization of nanomaterials.

Gold nanorods can also absorb NIR light and convert it to heat for application in photothermal therapy. Chang et al.^[^^39^^]^ selected thiolated chitosan to replace CTAB in gold nanorods because of its biocompatibility. The researchers then conjugated the chitosan-based GNRs with a disiloganglioside (GD2) monoclonal antibody, which could lead to the functionalized nanomaterials being endocytosed into the stNB-V1 neuroblastoma cells. Exposure to an NIR laser at 808 nm demonstrated that photo-thermal therapy could destruct the stNB-V1 cells by calcein AM staining.

## Application of Functionalized Gold Nanorods in Tumor Imaging and Therapy

Nowadays, more and more functionalized gold nanorods are attracting the attention of researchers because of their potential application in tumor imaging and therapy. Several of these applications are reviewed in this paper.

### Folic acid-conjugated silica-modified gold nanorods for CT imaging and therapy

**Figure 3:**
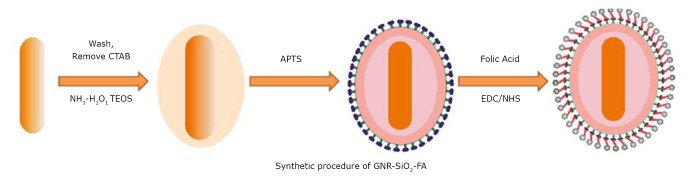
Synthetic procedure of GNR-SiO_2_-FA. Reprinted with permission from [40], Huang P, Bao L, Zhang CL, et al. Folic acid-conjugated Silica-modified gold nanorods for X-ray/CT imaging-guided dual-mode radiation and photo-thermal therapy. Biomaterials 2011; 32: 9796-9809.

Huang et al.^[^^40^^]^ utilized silica to replace CTAB molecules on the surface of GNRs by the classic Stöber method, thus eliminating cytotoxicity and improving biocompatibility. Folic acid was anchored on the surface of the silica-modified GNRs to form a multifunctional GNR-SiO_2_-FA nanoprobe (**Figure 3****)**.

**Figure 4:**
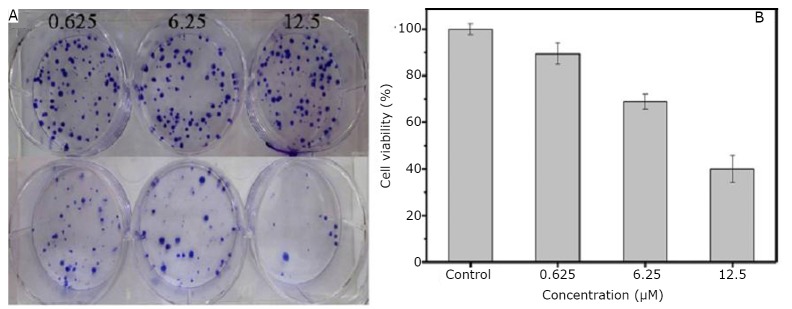
Proliferation (A) and cell viability (B) of MGC803 cells incubated with 100 µL of GNR-SiO_2_-FA in varying concentrations for 24 h at 6 Gy of X-ray irradiation. In the photographs (A), the top column of the cells did not receive irradiation; the bottom column of the cells received irradiation. Reprinted with permission from [40], Huang P, Bao L, Zhang CL, et al. Folic acid-conjugated Silica-modified gold nanorods for X-ray/CT imaging-guided dual-mode radiation and photo-thermal therapy. Biomaterials 2011; 32: 9796-9809.

The prepared nanoprobes displayed highly selective targeting, enhanced RT effects, and excellent PTT effects on the gastric cancer cells; the nanoprobes also exhibited strong X-ray attenuation in *in vivo* X-ray/CT imaging. In their experiment, Huang et al. incubated the MGC803 cells with varying concentrations of GNR-SiO_2_-FA to determine whether GNR-SiO_2_-FA can enhance the radiosensitivity of the MGC803 cells to different doses of X-ray irradiation. **Figure 4A** shows the proliferation of the MGC803 cells with and without irradiation. The number of colonies decreased with the increase in the concentration of GNR-SiO_2_-FA. Cell viability was normalized to that of the control cells (no drug and no irradiation) as shown in **Figure 4B**.

**Figure 5:**
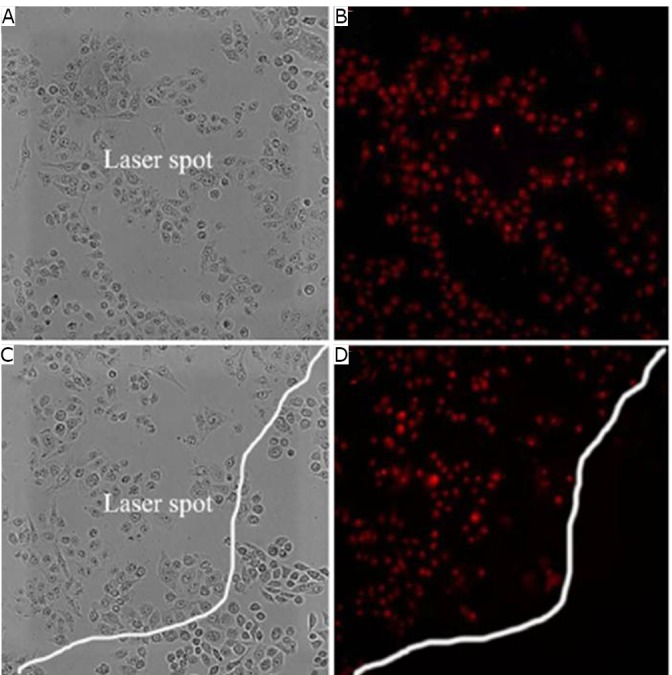
Effects of photothermal therapy on MGC803 cells incubated with 12.5 µM of GNR-SiO_2_-FA for 24 h at 37°C in the dark prior to irradiation for 3 min with an nm laser. A and B: MGC803 cells at the center of the laser spot. C and D: MGC803 cells on the boundary of the laser spot. A and C: Bright field, B and D: Fluorescence field. Reprinted with permission from [40], Huang P, Bao L, Zhang CL, et al. Folic acid-conjugated Silica-modified gold nanorods for X-ray/CT imaging-guided dual-mode radiation and photo-thermal therapy. Biomaterials 2011; 32: 9796-9809.

The researchers also tested the effects of GNR-SiO_2_-FA nanoprobes on photothermal therapy. **Figure 5A******and******5B****)** indicated that the MGC803 cells at the center of the laser spot were killed by the GNR-SiO_2_-FA upon irradiation. The cells on the boundary of the laser spot did not receive irradiation; they appeared spindle-like and did not exhibit red fluorescence as shown in **Figure 5C******and******5D****)**.

**Figure 6:**
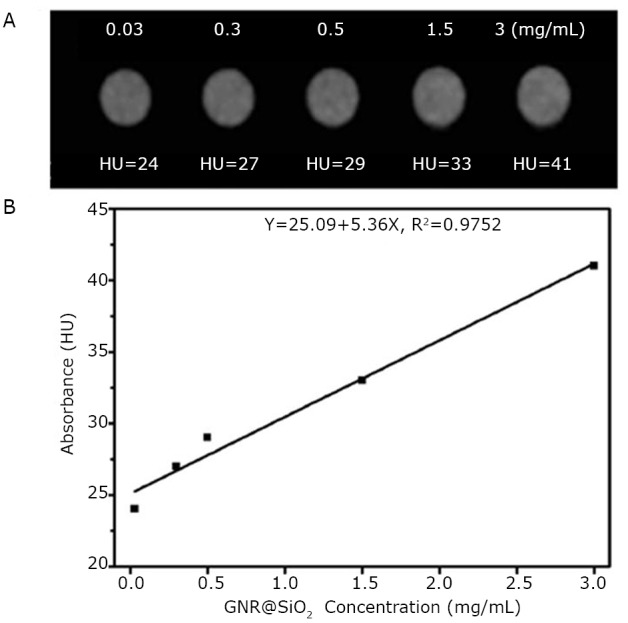
A: *In vitro* CT images of GNR-SiO_2_ suspended in PBS. The concentration (mg/mL) of each sample is provided at the top of the respective images. (B) CT attenuation (HU) plot of GNR-SiO_2_ at various concentrations in the range of 0.03 to 3 mg/mL. Reprinted with permission from [40], Huang P, Bao L, Zhang CL, et al. Folic acid-conjugated Silica-modified gold nanorods for X-ray/CT imaging-guided dual-mode radiation and photo-thermal therapy. Biomaterials 2011; 32: 9796-9809.

**Figure 7:**
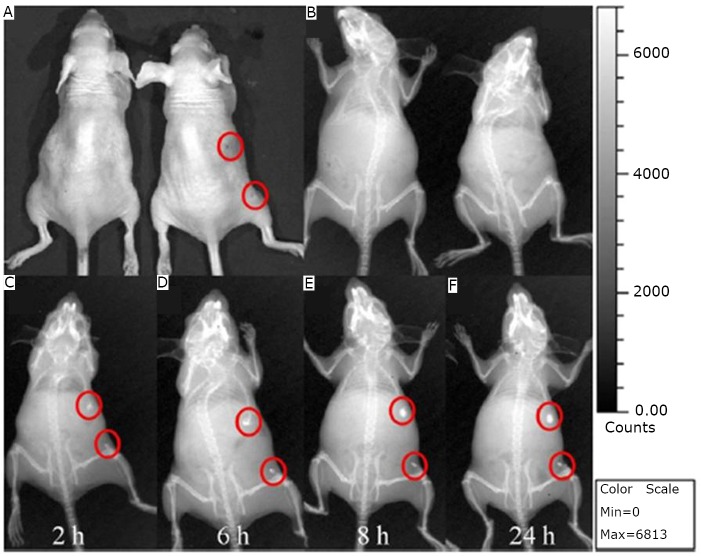
*In vivo* X-ray imaging of mice after subcutaneous injection without (left) and with GNR-SiO_2_-FA (right) at different time points. A: Photograph of mice; B: X-ray image at 0 h; C: X-ray image at 2 h; D: X-ray image at 6 h; E: X-ray image at 8 h; F: X-ray image at 24 h. Reprinted with permission from [40], Huang P, Bao L, Zhang CL, et al. Folic acid-conjugated Silica-modified gold nanorods for X-ray/CT imaging-guided dual-mode radiation and photo-thermal therapy. Biomaterials 2011; 32: 9796-9809.

**Figure 8:**
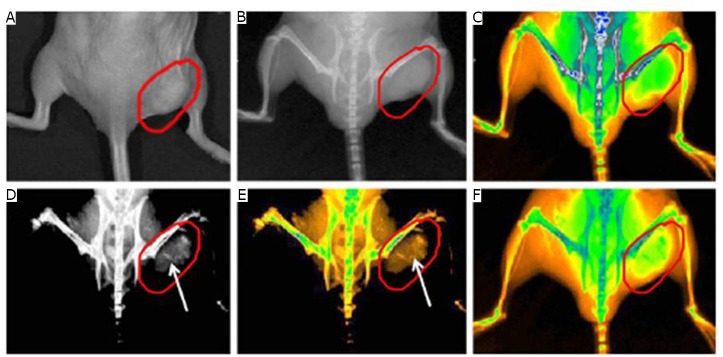
Real-time *in vivo* X-ray images after intravenous injection of GNR-SiO_2_-FA into nude mice at different point in time. A: Photograph of the tumor tissue; B: X-ray image at 0 h; C: X-ray image at 0 h (in color); D: X-ray image at 12 h; E: X-ray image at 12 h (in color); F: X-ray image at 24 h (in color). Reprinted with permission from [40], Huang P, Bao L, Zhang CL, et al. Folic acid-conjugated Silica-modified gold nanorods for X-ray/CT imaging-guided dual-mode radiation and photo-thermal therapy. Biomaterials 2011; 32: 9796-9809.

Aside from the abovementioned excellent effects, Huang et al.’s study^[^^40^^]^ showed that GNR-SiO_2_ could be utilized as a positive X-ray or CT imaging contrast agent. **Figure 6A** indicates that CT intensity continuously increased as the concentration of GNR-SiO_2_ increased. Moreover, the two had a well-correlated linear relationship as shown in **Figure 6B**. They also demonstrated the feasibility of GNR-SiO_2_-FA in *in vivo* X-ray imaging by injecting 5 mg and 1 mg of GNR-SiO_2_-FA into the back and right rear flank area of mice, respectively. As shown in **Figure 7**, the subcutaneous injection sites displayed a much more enhanced positive contrast than the other soft tissues. By lengthening the injection time, positive contrast became more and more clear. In addition, the researchers employed X-ray imaging to monitor the tumor tissue-targeting ability of GNR-SiO_2_-FA during blood circulation. **Figure 8** shows real-time *in vivo* X-ray images of nude mice after being injected with GNR-SiO_2_-FA at different points in time. The results showed that gastric cancer cells were targeted by GNR-SiO_2_-FA with high specificity.

### RGD-conjugated dendrimer-modified GNRs for tumor photothermal therapy

The arginine-glycine-aspartic acid (RGD) short peptides can specifically bind with integrin α_v_β_3_, which is important and over-expressed in sprouting tumor vessels and most tumor cells. To this end, Li et al.^[^^24^^]^ employed RGD to replace CTAB molecules on the surface of GNRs. The RGD-conjugated dendrimer-modified GNRs exhibited highly selective targeting and destructive effects on the cancer cells and solid tumors under near-infrared laser irradiation.

**Figure 9:**
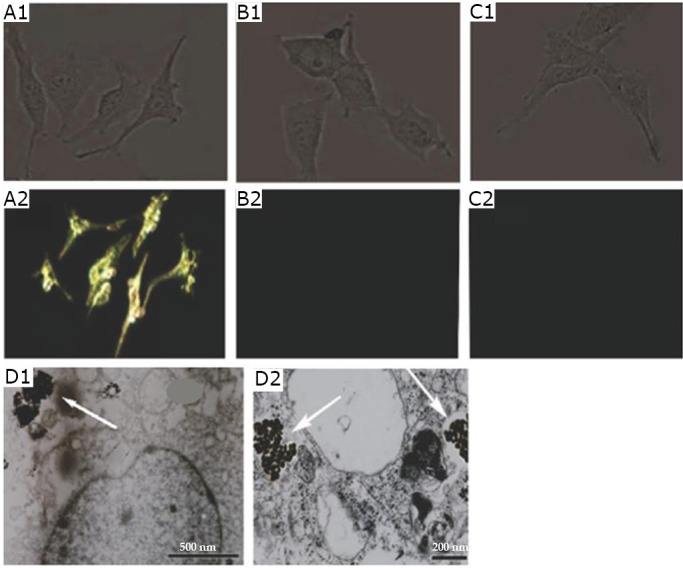
Light scattering images and intracellular location of RGD-dGNRs. Reflective mode dark-field images and bright images of RGD-dGNRs (A), dGNRs (B), and free peptide RGD-dGNRs (C) after incubation with A375 cells for 30 min at room temperature. The images were acquired with a Zeiss Axioscope2 microscope imaging system. A1: Bright-field image of A375 cells incubated with RGD-dGNRs exhibiting cell shapes. A2: Dark-field image of A375 cells incubated with RGD-dGNR nanoprobes exhibiting golden color. B1: Bright-field image of A375 cells incubated with dGNRs exhibiting cell shapes. B2: Dark-field image of A375 cells incubated with RGD-dGNR nanoprobes exhibiting black color. C1: Bright-field image of A375 cells incubated with free peptide and RGD-dGNRs exhibiting cell shapes. C2: Dark-field image of melanoma A375 cells incubated with free peptide and RGD-dGNRs, exhibiting black color. D1 and D2: TEM images of RGD-dGNRs (arrow) in the cytoplasm of melanoma A375 cells, left scale bar, 500 nm; right scale bar, 200 nm. Reprinted with permission from [24], Li Z, Huang P, Zhang X, et al. RGD-conjugated dendrimer-modified gold nanorods for *in vivo* tumor targeting and photothermal therapy. Mol Pharm 2010; 7: 94-104.

Li et al.^[^^24^^]^ also evaluated the specificity and sensitivity of RGD-dGNR nanoprobes in tumor cell targeting. Melanoma A375 cells incubated with RGD-dGNR nanoprobes exhibited a strong golden color, whereas the melanoma A375 cells incubated with free RGD peptides and pre-incubated with free RGD peptides did not exhibit a golden color. RGD-dGNR nanoprobes were located on the surface and in the cytoplasm of the melanoma A375 cells (**Figure 9**). This finding suggests that RGD-dGNR nanoprobes can specifically target melanoma A375 cells.

**Figure 10:**
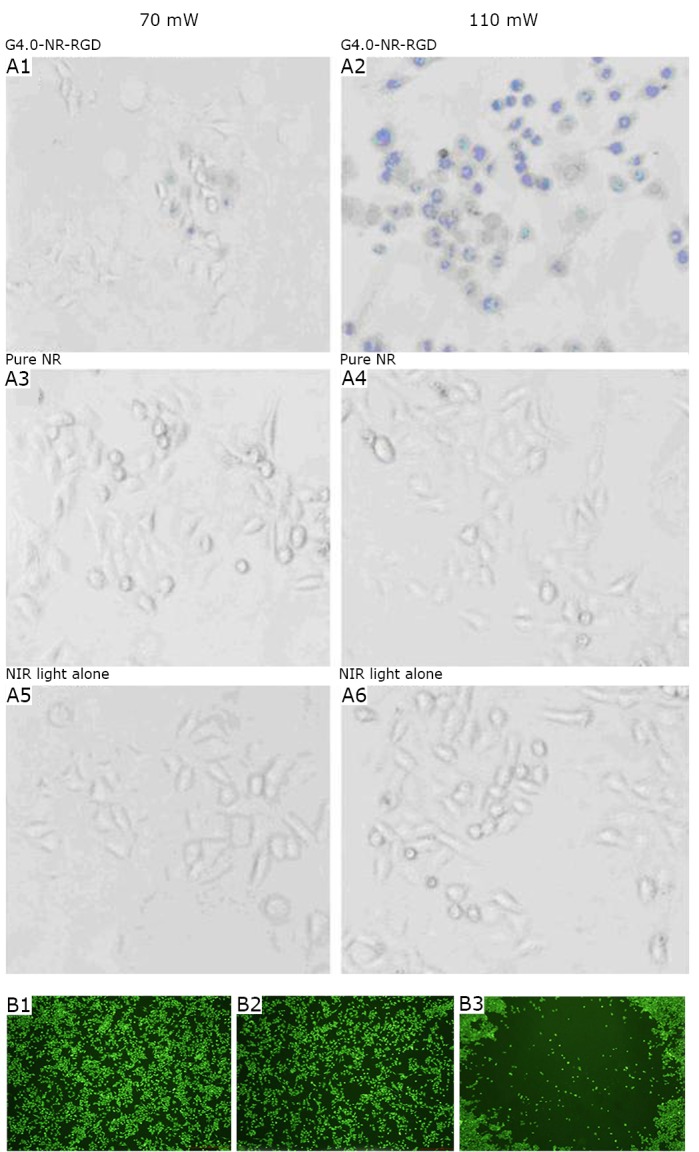
A: The cells treated with G4.0-NR-RGD conjugation start to be injured at 70mW (9 W/cm^2^) and are obviously injured at 110mW (14 W/cm^2^). A1 and A2: The cells treated with pure gold nanorods; A3 and A4: The cells are not affected; A5 and A6: NIR light alone. B: Cell viability assessed via calcein staining. B1: No dead cells in the test group with 0.1 µg/mL of RGD-dGNRs. B2: No dead cells in the test group with 5 µg/mL of RGD-dGNRs. B3: Cell death and disappearance within the spot in the test group with 100 µg/mL of RGD-dGNRs. Reprinted with permission from [24], Li Z, Huang P, Zhang X, et al. RGD-conjugated dendrimer-modified gold nanorods for *in vivo* tumor targeting and photothermal therapy. Mol Pharm 2010; 7: 94-104.

They also tested the viability of the cells at 110 mW laser irradiation for 4 min (**Figure 10 A**). The amount of destroyed cells increased as the amount of RGD-dGNR nanoprobes in the medium increased. When the concentration of the RGD-dGNR nanoprobes reached 100 µg/mL, all the cancer cells within the laser spots were killed as shown in **Figure 10** **B**. The optimal photothermal therapeutic concentration was 100 µg/mL for *in vitro* cancer cells.

**Figure 11:**
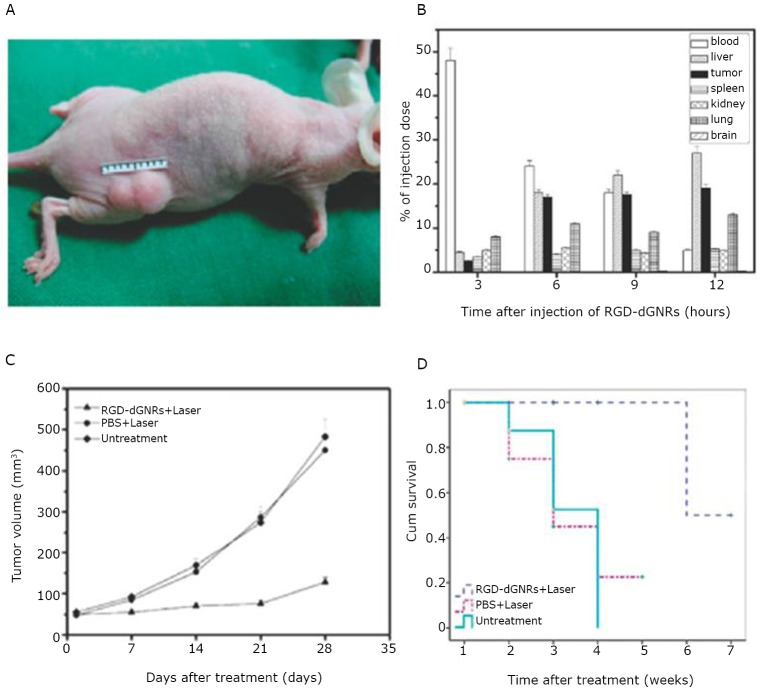
Melanoma animal models, biodistribution of RGD-dGNRs, and survival data analysis of control and test group. A: A375 melanoma mouse models. The tumor size can be calculated as ab^2^/2 (a represents the longer dimension and b represents the shorter dimension of the tumor). B: Biodistribution of RGD-dGNRs in mice after intravenous injection. After injection, the gold amounts in the tissue samples were evaluated through ICP mass spectrometry (*n*=3). C: Tumor size at different points after the irradiation of mice treated with RGD-dGNRs and NIR laser (group 1); PBS and NIR laser (group 2) or untreated control (group 3), *P*<0.05 for group 2 or group 3 *vs.* group 1. D: Kaplan-Meier curve of the test group and control group, *P*=0.006. Reprinted with permission from [24], Li Z, Huang P, Zhang X, et al. RGD-conjugated dendrimer-modified gold nanorods for *in vivo* tumor targeting and photothermal therapy. Mol Pharm 2010; 7: 94-104.

**Figure 11A** shows mouse models with melanoma A375 cells. The nanoprobes in the tumor tissues increased gradually as time increased (**Figure 11B****)**. Seventeen percent of the RGD-dGNR nanoprobes accumulated in the local tumor tissues at 6 hours after injection. Thus, six hours was selected as the optimal time to begin the NIR laser irradiation on the tumor locations. As the laser irradiation time and mouse breeding time increased, the size of the tumor in the test group became smaller and smaller. On the contrary, the tumor grew larger and larger in the control group (**Figure 11C**). The Kaplan-Meier curve (**Figure 11D**) suggested that the therapy based on nanoprobe injection and NIR laser irradiation can markedly increase the survival time of mice with tumors.

**Figure 12:**
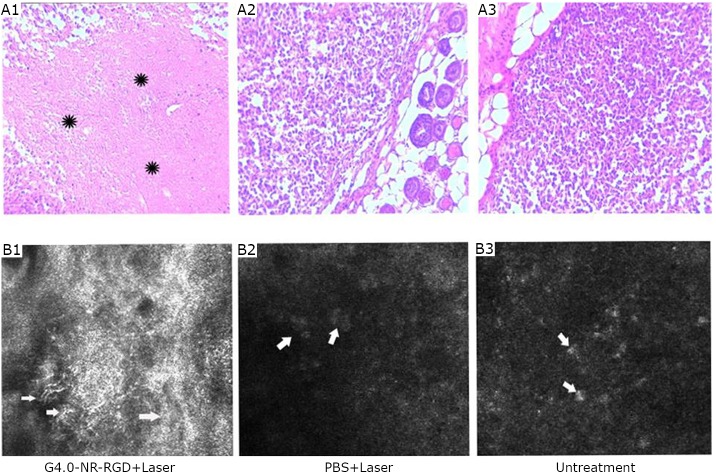
RCM images and histological images of the tumor seven weeks after treatment. A: RCM images (0.25 mm × 0.25 mm) of the tumor were acquired with a VivaScope 3000 microscope (Lucid) using a diode laser with a wavelength of 830 nm and an objective lens with 30× numerical aperture (NA) and 0.9 water immersion. A1: Arrows point to the numerous gossamer-like collagen bundles and branch-like collagens. A2 and A3: Arrows point to the reflective melanoma cells existing in a low-density and sparse cluster. B: Corresponding histological images (20×) of the tumor after H&E staining as well as laser irradiation in the group of RGD-dGNRs (B1), group of PBS after laser irradiation (B2), and the untreated control group (B3). B1: Scar-like structure containing numerous collagen bundles in the tumor location. B2: Existence of numerous tumor cells. B3: Existence of numerous tumor cells. Reprinted with permission from [24], Li Z, Huang P, Zhang X, et al. RGD-conjugated dendrimer-modified gold nanorods for *in vivo* tumor targeting and photothermal therapy. mol pharm 2010; 7: 94-104.

Li et al.’s study also investigated the potential mechanism of RGD-dGNR nanoprobe-based photothermal therapy by real-time reflectance confocal microscopy (RCM). The RCM images of the tumors in the test group displayed plenty of gossamer-like collagen bundles and branch-like collagens (white arrow in **Figure 12** **A1**). Numerous reflective melanoma cells also existed in the control group (**Figure 12** **A1 and A2**). In the test group (**Figure 12** **B1**), a scar-like structure containing numerous collagen bundles was observed. Numerous tumor cells were also observed in the control group (**Figure 12** **B2 and B3)**.

**Figure 13:**
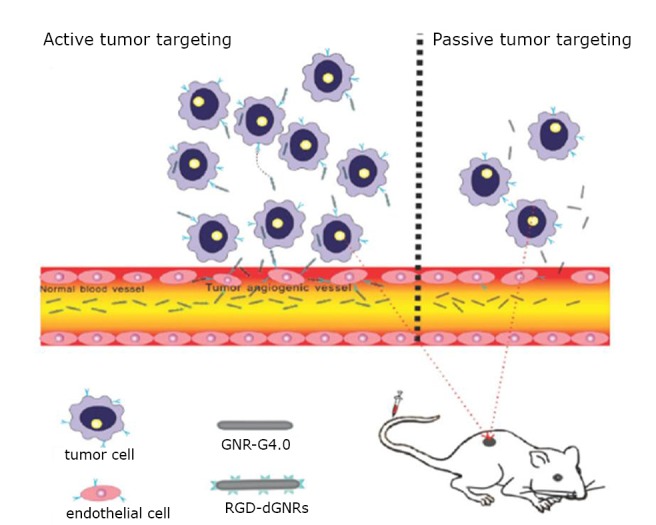
Accumulation of gold nanorods in the tumors through both EPR and active binding. Reprinted with permission from [24], Li Z, Huang P, Zhang X, et al. RGD-conjugated dendrimer-modified gold nanorods for *in vivo* tumor targeting and photothermal therapy. mol pharm 2010; 7: 94-104.

All the above results confirmed that RGD-dGNR nanoprobes have active targeting abilities and exhibit selective destructive effects on targeted cancer cells under NIR laser irradiation. The results also suggested three possible therapeutic mechanisms, one of which is shown in **Figure 13**.

### Chiral gold nanoflowers for tumor photothermal therapy

**Figure 14:**
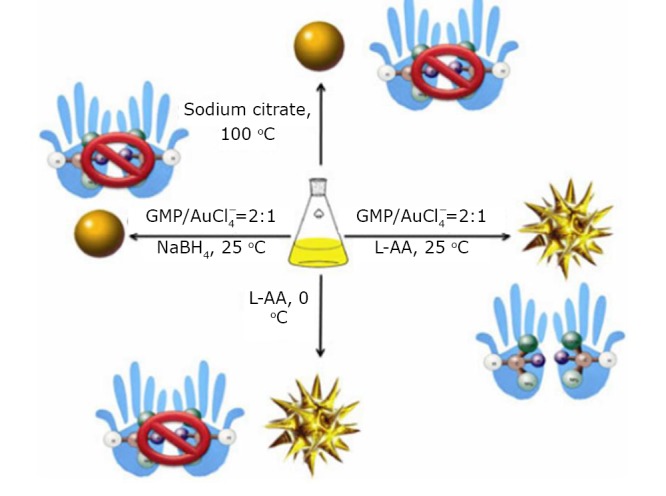
Schematic of different reaction conditions used to control the morphology and chirality of gold nanocrystals. Reprinted with permission from [41], Huang P, Pandoli O, Wang X, et al. Chiral guanosine 5’-monophosphate-capped gold nanoflowers: Controllable synthesis, characterization, surface-enhanced Raman scattering activity, cellular imaging and photothermal therapy. Nano Res 2012: 5: 630-639.

**Figure 15:**
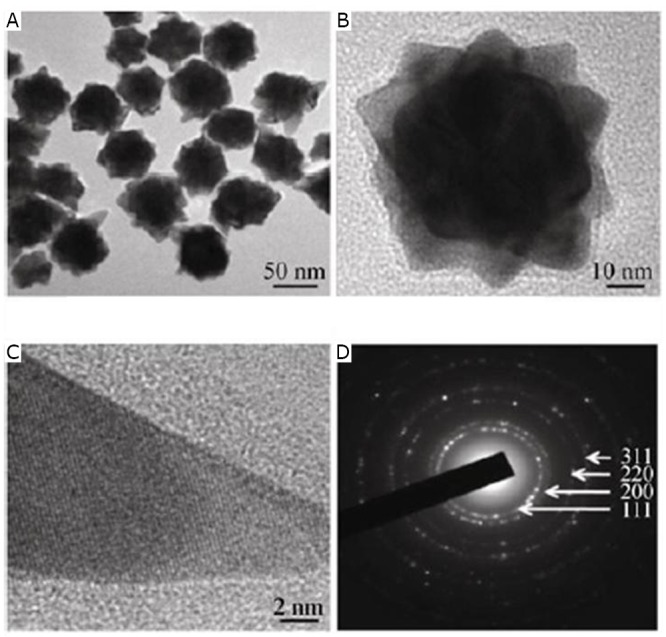
Gold nanoflowers synthesized by the reduction of L-AA at 1 h time point. A: TEM image, B: HRTEM image of an individual nanoflower, C: HRTEM image of an individual petal tip, D: corresponding SAED pattern. Reprinted with permission from [40], Huang P, Bao L, Zhang CL, et al. Folic acid-conjugated Silica-modified gold nanorods for X-ray/CT imaging-guided dual-mode radiation and photo-thermal therapy. Biomaterials 2011; 32: 9796-9809.

Recently, nanogold species with various shapes have been developed because of their surface- and morphology-dependent properties. Huang et al.^[^^41^^]^ studied the simple one-pot green synthesis of chiral GNFs with abundant petal-shaped tips by chloroauric acid with L-ascorbic acid (L-AA) as a reducing agent in the presence of a 5^’^-GMP nucleotide. As shown in **Figure 14**, when the reducing agent was L-AA, the obtained nanocrystals were flower-like in shape and produced an LSPR band over 570 nm. The synthesized nanocrystals were quasi-spherical, consisting of a solid core with many (>10) short, irregular, and obtuse branches (**Figure 15A**). **Figure 15B** displays an individual flower-like nanocrystal with highly asymmetrical metallic surface morphology. **Figure 15C** shows a petal-shaped tip as a single-crystalline protrusion from the core of the nanoflower. The corresponding selected area electron diffraction (SAED) pattern of the area containing many GNFs is shown in **Figure 15D**. The figure also reveals that the nanoflowers are crystalline and randomly oriented.

**Figure 16:**
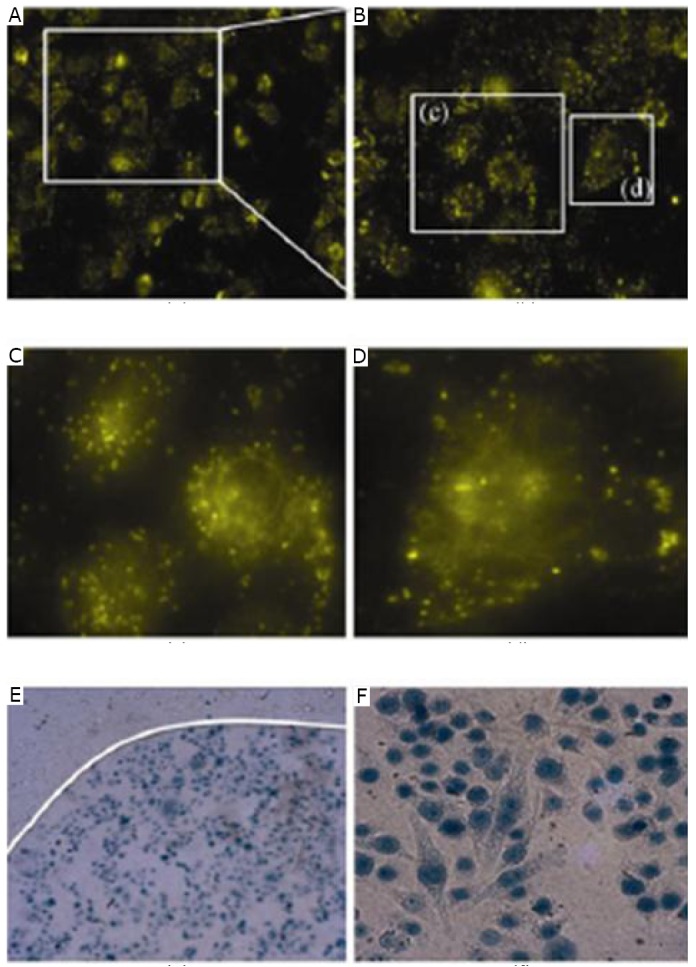
Dark-field scattering images. A: Low-magnification image; B-D: High-magnification images of the MGC803 cells incubated with 50 µmol/L of GNFs for 2 h and monitored by dark-field microscopy; E, Effect of photothermal therapy on MGC803 cells at the boundary of the laser spot incubated with 10 µmol/L GNFs for 24 h at 37°C in the dark prior to irradiation for 5 min with a 632.8 nm He–Ne laser with energy density of ~30 mW/cm^2^; F: MGC803 cells on the laser spot. Reprinted with permission from [40], Huang P, Bao L, Zhang CL, et al. Folic acid-conjugated Silica-modified gold nanorods for X-ray/CT imaging-guided dual-mode radiation and photo-thermal therapy. Biomaterials 2011; 32: 9796-9809.

To analyze further the biomedical application of chiral GNFs, Huang et al.^[^^41^^]^ also measured the dark cytotoxity of the cells. The results showed that chiral GNFs were non-cytotoxic and had excellent biocompatibility with MGC803 cells within the concentration range of 0 to 200 µmol/L. To determine the intracellular uptake of chiral GNFs, dark-field scattering imaging was performed on the MGC803 cells (**Figure 16**). As shown in **Figure 16 (C and D)**, the cells displayed an intense homogeneous cytoplasmic golden color around the nucleus. This observation indicated that GNFs accumulated in the cells. Through irradiation by a 632.8 nm He-Ne laser, the GNFs could convert the absorbed photons into thermal energy for tumor cell PTT. On the boundary of the laser spots, the cells without irradiation were in good physiological state and appeared spindle-like (**Figure 16E**). On the laser spot, all the cells were killed. The dead cells exhibited blue nuclei (**Figure 16F**). The above results suggested that the as-synthesized GNFs with good biocompatibility can be utilized in cellular dark-field imaging and photothermal therapy.

Aside from the abovementioned applications, gold nanorods can also be utilized in optoacoustic imaging. Several groups^[^^42^^-^^44^^]^ have utilized GNRs as contrast agents, and they concluded that GNRs are highly sensitive^[^^42^^]^ and can be used in multispectral optoacoustic imaging^[^^43^^]^.

## Challenges and Prospects

The nascent field of GNR-based nanotechnology presents an exciting vision of the future. The ultimate goal of this field is to design and construct functional GNR materials and nanodevices at the nanoscale level. Exploring the biomedical engineering applications of GNRs lead to the in-depth understanding of the fundamental laws and principles of biochemistry, biocompatibility, molecular biology, and materials science. This new understanding will enable the design and fabrication of a vast variety of nanoprobes and nanodevices for tumor molecular imaging and targeted therapy. The surface functionalization of GNRs enhances their biocompatibility and clarifies their bio-distribution. GNR-based nanoprobes and nanodevices will contribute to clinical applications in the future because they exhibit excellent application potential.

## Conclusion

In this paper, we summarized several recent techniques for surface modification of gold nanorods. These techniques are useful for the improvement of the properties of gold nanorods and for solving the problems caused by CTAB. The different methods have different advantages. Magnetism is the preponderance of iron oxide-coated gold nanorods, and a silica shell can provide better biocompatibility. Several studies showed that silica-coated gold nanorods could amplify the photoacoustic response without significantly changing the optical absorption of nanorods other than shifting it slightly to red^[^^45^^]^. PEGylation allowed the bioconjugation of the nanorods in solution and the processing of nanorods into well-ordered films^[^^9^^]^. All these methods not only solved the problem of CTAB being a toxic surfactant in the synthesis of gold nanorods, but also provided more techniques to optimize surface functionalization. The unique properties of gold nanorods make them promising materials for biomedical application.
